# Evaluation on the Performance of Hydraulic Bitumen Binders under High and Low Temperatures for Pumped Storage Power Station Projects

**DOI:** 10.3390/ma15051890

**Published:** 2022-03-03

**Authors:** Changgen Zou, Zhao Hua, Liantong Mo, Cong Qi, Zhixin Liu, Yanjun Xie, Hao Yu, Juntao Ke

**Affiliations:** 1 State Key Laboratory of Silicate Materials for Architectures, Wuhan University of Technology, Wuhan 430070, China; zoucg@whut.edu.cn (C.Z.); 303621@whut.edu.cn (H.Y.); 2 China Gezhouba Group Municipal Engineering Co., Ltd., Yichang 443002, China; huazhao@gzbjtszjsgcyx.wecom.work; 3 Shandong Haiyun Asphalt Co., Ltd., Binzhou 371600, China; cong.qi@chambroad.com; 4 China Water Northeastern Investigation Design and Research Co., Ltd., Changchun 130021, China; szn-mjy-2005@163.com; 5 Gezhouba Group Testing Co., Ltd., Yichang 443002, China; xieyanjun@cljcs.wecom.work; 6 Hubei Yichang Dingcheng Engineering Testing Co., Ltd., Yichang 443002, China; kejuntao@hbsycsdcgcjsf.wecom.work

**Keywords:** hydraulic bitumen, rheological properties, creep resistance, tensile failure strain, relaxation modulus, flow slope, failure temperature

## Abstract

The high and low-temperature performance of five hydraulic bitumen binders was evaluated using the dynamic shear rheometer (DSR) test, infrared spectrum test and direct tensile (DT) test. These hydraulic bitumen binders were respectively applied for several pumped storage power stations (PSPS) projects that were constructed or under construction. In order to relate the bitumen performance to the mixture performance, the slope flow test, three-point bending test and thermal stress restrained specimen test were carried out on hydraulic asphalt mixtures. The test results indicated the DSR rheological master curves can well distinguish the difference of each bitumen binder as well as the effect of polymer modification. Phase angle master curves, black diagrams and infrared spectra all indicated that several penetration-grade hydraulic bitumen binders were not virgin bitumen binders but were modified with relatively lower SBS polymer content when compared with traditional SBS-modified bitumen. When selecting the commonly used Karamay SG70 hydraulic bitumen as a reference, the normal SBS-modified bitumen was superior to other bitumen in terms of low- and high-temperature performance. Several slightly SBS-modified bitumen binders did not always show consistent results, which indicated that slightly modified bitumen may not really have the desired performance as expected. Therefore, SBS-modified bitumen will be more promising when dealing with extremely low or high temperatures. Bitumen performance was well compared with the mixture performance by using the bitumen creep, relaxation and tensile failure strain corresponding to the asphalt concrete slope flow, the maximum bending strain and the failure temperature, respectively. Compared with the traditional penetration, softening point and ductility test, it indicated that the DSR rheological test, creep test, direct tensile test and stress relaxation test can be used as more powerful tools for the characterization and optimization of hydraulic bitumen binders.

## 1. Introduction

China is currently striving to meet a grand goal: to peak its carbon dioxide emissions before 2030 and achieve carbon neutrality before 2060. For this reason, the construction of pumped storage power stations (PSPSs) in China has entered a stage of rapid development to improve and adjust the state power source structure. According to the Medium- and Long-term Development Plan for Pumped Storage (2021–2035) issued by the Chinese National Energy Administration, about 550 PSPSs are planned for construction in the next fifteen years. The PSPS installed capacity had reached 3.249 million kilowatts by the end of 2020 in China. More than 100 PSPSs are currently under construction [1,2].

Because of the excellent anti-seepage and deformation properties, hydraulic-rolled asphalt concrete anti-seepage facing is an important form of anti-seepage structure for pumped storage reservoirs. At present, a simple panel structure consisting of an asphalt concrete leveling layer with a thickness of 100 mm, an asphalt concrete impermeable layer with a thickness of 100 mm and an asphalt mastic sealing layer with a thickness of 2 mm is mainly applied in China for the consideration of cost and the ease of construction. Among the three layers, the leveling layer is made by open-graded asphalt concrete with an air void content between 10% and 15%, and the impermeable layer is made by dense-graded asphalt concrete with an air void content below 3%, while the confining/sealing layer is made using asphalt mastic that usually consists of 35% bitumen and 65% filler by weight. The asphalt concrete impermeable layer is usually regarded as the most important structural layer, which requires good anti-seepage, thermal and water stability, crack resistance, durability and workability during construction [3,4]. The selection of raw material and the mix design of the dense-graded asphalt concrete thus become very important, since they are closely related to the above functions and the performance of the impermeable layer [5,6].

Because of the main concern for the durability of the impermeable layer in China, the hydraulic bitumen used in previous projects were specially produced for each project and usually had higher quality requirements compared with the commonly used road paving bitumen [6]. Although hydraulic bitumen is classified according to the conventional penetration grade in the current Chinese specification, significant improvements on some technical requirements, for example, the loss of mass, residual penetration, ductility and the increase of the softening point after the thin film oven test (TFOT), were made compared to road-paving bitumen. As a result, the traditional road-paving bitumen cannot fulfill the specifications. This also limits the scope of the bitumen selection and restricts it to a few designated bitumen manufacturers. In the past, virginal bitumen was firstly used for the anti-seepage panel of the PSPSs. However, the service performance was not satisfactory due to the slope flow at high temperature and the cracking at low temperature. Because the crude oil and processing techniques used for hydraulic bitumen are currently experiencing a fundamental change in China, the application of polymer-modified bitumen is becoming the trend for PSPSs. Polymer-modified bitumen will be more promising when dealing with extremely low and high temperatures [7,8,9]. The properties of hydraulic bitumen are evaluated mainly based on traditional penetration, the softening point and ductility. It should be noted that the above three indices cannot well reflect the real performance of the bitumen used in the field. This has been demonstrated in the field of asphalt pavements, which usually has a typical three-layer structure, including the surface wearing course, the intermediate layer and the base course layer from top to down, respectively [10,11]. For this reason, rheological analysis was widely used for road paving bitumen by means of the dynamic shear rheometer (DSR), the bending beam rheometer (BBR) and the direct tensile tests [12,13]. Among these methods, the DSR test can provide abundant information on rheological properties in terms of the complex modulus (G*), the phase angle (δ), the rutting factor (G*/sinδ), the fatigue factor (G*sinδ), the creep compliance and the stress relaxation. The results of the DSR testing, especially the multiple stress creep recovery (MSCR) tests, can well distinguish the rheological properties of bitumen at high temperature. The non-recoverable creep compliance (Jnr) and the recovery percentage (R%) are recommended to describe the high-temperature properties of different types of bitumen. The stiffness modulus and stiffness dissipation rate (m-value) obtained from the BBR test are the main parameters for characterizing the low-temperature performance of bitumen. Furthermore, the direct tensile test at extremely low temperature is used to determine the critical cracking temperature of bitumen [14,15,16]. The advanced rheological tests, as mentioned above, have not been applied for the characterization and optimization of hydraulic bitumen binders.

Hydraulic-rolled asphalt concrete is widely used in core walls in dams; however, its application in anti-seepage facings is limited to approximately 12 PSPSs in China [17,18,19]. Therefore, the knowledge and experience on the rolling asphalt concrete facings of PSPSs is insufficient in the matter of material selection and optimization, mixture design and construction. It is difficult to deal with the great demand of PSPSs as planned in China when considering different geographical zones. With respect to hydraulic bitumen, little research has been done on the rheological performance analysis. Furthermore, the service condition, mixture design and the construction of hydraulic-rolled asphalt concrete are different from those of asphalt pavement concrete. The crude oil and processing techniques used for hydraulic bitumen are changing in China, and the commonly accepted and used hydraulic bitumen, for example, Karamay bitumen, cannot meet the current great demand. Various virgin bitumen and polymer-modified bitumen produced from different manufacturers have been used in recent PSPS projects. Therefore, it is necessary to gain insight into the fundamental rheological characteristics of these hydraulic bitumen binders that were actually applied in field and to establish a performance-based specification for bitumen selection and optimization.

In this study, five different hydraulic bitumen binders obtained from various PSPS projects that have been constructed or were under construction were selected for comprehensive rheological evaluation. Focus was on the high- and low-temperature properties, which were mainly characterized by means of the DSR and DT tests. The rheological master curves of these hydraulic bitumen binders were built by considering a wide range of test temperatures and frequencies. The creep test at high temperature and the stress relaxation test at low temperature, as well as direct tensile test at extremely low temperature, were performed. Furthermore, in order to relate the rheological properties of hydraulic bitumen binders with the performance of the prepared asphalt mixture, the slope flow test, three-point bending test and thermal stress restrained specimen test were carried out on hydraulic asphalt mixtures. The test data obtained from the hydraulic bitumen and its mixtures were aimed at establishing a benchmark for the selection and development of better hydraulic bitumen based on the rheological performance.

## 2. Materials and Methods

### 2.1. Materials

Five different hydraulic bitumen binders, including Karamay SG70 bitumen, Jingbo SG70 bitumen, Jingbo SBS polymer-modified bitumen (PMB), Liaohe SG90 bitumen and Jurong SG90 bitumen, were used in this study. Among these bitumen binders, Karamay SG70 is the commonly accepted and used hydraulic bitumen, produced by Karamay Petrochemical Co., Ltd. (Beijing, China) of CNPC. Jingbo SG70 bitumen and Jingbo SBS polymer-modified bitumen, produced by Shandong Chambroad Petrochemicals Co., Ltd. Were used for the Yimeng PSPS project in Shandong province. The Jingbo SG70 bitumen was used for the asphalt concrete leveling layer, while the Jingbo PMB was used for asphalt concrete impervious layer. Liaohe SG90 bitumen, which was produced by Liaohe Petrochemical Co., Ltd. (Panjin, China) of CNPC, was used for the core wall of the Dunhua project in Jilin province [20]. Jurong SG90 bitumen, which was produced by Liaohe Petrochemical Co., Ltd. Of CNPC, was used for the Jurong PSPS project in Jiangsu province. The basic properties of the five hydraulic bitumen binders as mentioned above are shown in Table 1. Three performance grades were considered, including hydraulic bitumen penetration 70 (SG70), penetration 90 (SG90) and SBS (I-C) polymer-modified hydraulic bitumen. These different bitumen binders were selected for different projects according to the service temperatures of each project. Polymer-modified hydraulic bitumen was selected in the case that virgin bitumen could not meet the requirements on extremely low- and high-temperature properties. In this study, all the test bitumen samples were original without being subjected to short- or long-term aging. Virgin bitumen is widely used as a hydraulic bitumen in China and the ductility is tested according to the Test Code for Hydraulic Asphalt Concrete, in which the test temperature was at 4 °C [21]. At present, polymer-modified bitumen is rarely used as a hydraulic bitumen, and the quality control is temporarily tested according to the Standard Test Methods of Bitumen and Bituminous Mixtures for Highway Engineering, in which the ductility for polymer-modified bitumen is done at 5 °C [22].

As indicated in Table 1, there was very limited difference between the penetration-grade SG70 and SG90 in the softening point, and thus the high-temperature performance cannot be well distinguished. Ductility is a method to measure the deformation capacity of the bitumen. The Karamay SG70 bitumen had the smallest ductility, followed by Jingbo PMB. This trend did not well reflect the performance advantage of the polymer-modified bitumen at low temperature. A similar result was also observed on the ductility after aging. Jingbo PMB did not exhibit a clear advantage in terms of ductility before and after aging. This strongly indicated that the quality and performance of the hydraulic bitumen was difficult to evaluate by using only the traditional penetration, softening point and ductility indices. The use of advanced rheological test methods was thus necessary for the characterization and optimization of hydraulic bitumen.

### 2.2. Test Methods

#### 2.2.1. Dynamic Shear Rheometer (DSR) Test

Bitumen is a viscoelastic material, and thus the temperature and rate/load time are two important factors that affect its rheological properties. In this study, a dynamic shear rheometer, DSR (MCR101, Anton Paar, Germany), was used to test the rheological parameters of the phase angle (δ) and the complex modulus (G*). The frequency sweep tests were performed at various temperatures including −10 °C, 0 °C, 20 °C, 30 °C, 40 °C, 50 °C, 60 °C and 70 °C. The frequency sweep ranged from 0.1 Hz to 50 Hz. The parallel plates with a diameter of 8 mm and a 2 mm gap were used for tests at temperatures below 25 °C, while 25 mm parallel plates and a 1 mm gap were used for tests at temperatures above 25 °C. The obtained rheological parameters of the phase angle and the complex modulus at various temperatures were used to construct the master curves based on the time–temperature superposition principle [23].

#### 2.2.2. Fourier Transform Infrared Spectroscopy (FTIR) Test

Fourier transform infrared spectroscopy (FTIR) analysis is a commonly used technology to monitor the molecular changes associated with bitumen oxidation and aging [24]. Carbonyl groups and sulfoxides are the major functional groups formed during bitumen oxidative aging. Both can be well identified by C=O at 1700 cm^−1^ and S=O at 1030 cm^−1^, respectively, in FTIR spectroscopy. For SBS polymer-modified bitumen binders, the SBS polymer consists of a polybutadiene segment in the middle with polystyrene blocks at the ends, which contains the C=C bonds in the unsaturated carbon chains and benzene rings, respectively. Two special absorption peaks at 966 cm^−1^ and 699 cm^−1^ can identify the polybutadiene (PB) and polystyrene (PS), respectively, and thus they can be used to characterize the existence of SBS in its modified asphalt binder with FTIR spectroscopy [25,26]. Furthermore, the degradation of the SBS polymer during binder aging can also be detected. In this paper, interest is focused on the detection of the existence of the SBS polymer, while the effect of aging will be analyzed in future studies.

Hydraulic bitumen has higher technical requirements compared with traditional road paving bitumen in China. This makes the traditional straight-run method difficult to produce qualified hydraulic bitumen. For this reason, some bitumen manufacturers have tended to add a relatively small amount of the SBS polymer into the base bitumen to adjust the high and low temperature properties. In order to test whether there is the presence of SBS in hydraulic bitumen or not, FTIR analysis was carried out on all of the five hydraulic bitumen binders, as mentioned before. The infrared analysis made use of the two characteristic absorption peaks at 966 cm^−1^ and 699 cm^−1^, which correspond to the functional groups of the polybutadiene (PB) and polystyrene (PS) of the SBS polymer, respectively. The FTIR spectra were recorded by a Fourier transform infrared spectrometer (Nicolet 6700, Thermo Electron Scientific Instruments Co., Madison, WI, USA) loaded with Omnic 8.2 software. A small pellet of bitumen sample was dissolved in an amount of carbon disulfide to prepare a 5 wt% concentration solution. Three drops of the solution were put onto a KBr slide, dried for the FTIR analysis and then scanned within the range from 4000 cm^−1^ to 400 cm^−1^.

#### 2.2.3. Multiple Stress Creep Recovery (MSCR) Test

The multiple stress creep recovery (MSCR) test was carried out to evaluate the high-temperature performance of the hydraulic bitumen. The test made use of 25 mm parallel plates with a 1 mm gap. Test temperature was determined at 70 °C, which is in agreement with the slope flow test of hydraulic asphalt concrete in the Chinese specification [21]. The slope flow test is used to evaluate the slope flowing resistance of asphalt concrete facing when subjected to extreme high temperature in summer. A one-second shear creep load was applied on the test sample, followed by a nine-second recovery. The first ten cycles of the creep and recovery were conducted under a shear load of 0.1 kPa. Then, a shear load of 3.2 kPa was applied on the same specimen for another 10 cycles. By calculating the average irrecoverable creep compliance (Jnr) and average recovery rate, the high-temperature performance of the five hydraulic bitumen binders was compared and analyzed. The Jnr and recovery percentage are determined as follows [11]:

(1) $$\mathrm{Jnr}=\frac{\mathrm{Nonrecoverable}\;\mathrm{strain}}{\mathrm{Stress}\;\mathrm{level}}$$

(2) $$\mathrm{recovery}\;\mathrm{percentage}=\frac{Recovered\;strain}{\mathrm{Maximum}\;\mathrm{strain}}\times 100$$

The Jnr is used to evaluate the binder’s contribution to the permanent deformation behavior, while the recovery percentage reflects the elasticity of hydraulic bitumen.

#### 2.2.4. Stress Relaxation Test

DSR was used to test the low-temperature stress relaxation of the five hydraulic binders. The test specimen made use of 8 mm parallel plates with a 2 mm gap. Two temperature conditions, including −10 °C and −25 °C, were considered to represent two different extreme low temperatures in south and north China, respectively. A constant strain was selected as 5% for −10 °C, while 0.5% for −25 °C. The strain was instantaneously applied, and the loading was continuously measured and recorded automatically over time. In total, a loading time of 600 s was applied.

#### 2.2.5. Direct Tensile (DT) Test

The direct tensile test was carried out by using a universal tensile testing machine. Three temperature conditions, including 0 °C, −15 °C and −25 °C, were considered according to the serving conditions of the hydraulic asphalt concrete facing. The temperature of 0 °C represented the frozen water temperature of the reservoir in winter. The temperatures of −15 °C and −25 °C represented two different extreme low temperatures that may be subjected in south and north China, respectively. The DT test was applied with a displacement-controlled mode of 1 mm/min. At extremely low temperature, bitumen is usually brittle and thus the DT test exhibits typical brittle failure, indicated by the stress linearly increasing with the strain and then the sudden break of the specimen. In this case, the strain at failure is corresponding to the strain at the maximum stress. At the test temperature of 0 °C and −15 °C, bitumen may become ductile, and the failure may become complex due to the necking effect during DT testing. The specimen may not fracture at the point of peak load, but after the peak load due to the necking effect. In this case, the stress at peak load is defined as the ultimate strength, and the strain at ultimate strength is not equal to the strain at failure. On the contrary, the strain at ultimate strength and the strain at failure are the same when bitumen is hard and brittle. The stiffness modulus of bitumen is defined by the slope of the proportional part of the stress–strain curve. For the ease of comparison, the bitumen stiffness at 0 °C was determined by using 5% strain and its corresponding stress; when the temperature was reduced to −15 °C, the bitumen stiffness was determined by using 2% strain and its corresponding stress; at −25 °C, the bitumen stiffness was obtained by using the ultimate strength divided by the corresponding strain, which was the strain at failure.

#### 2.2.6. Asphalt Concrete Test

In order to relate the rheological properties of the hydraulic bitumen binders with the performance of the prepared asphalt concrete, the performance tests of the hydraulic asphalt concrete were carried out by using the high-temperature slope flow test at 70 °C, the three-point bending (3PB) test at 2 °C and the thermal stress restrained specimen test (TSRST). These tests were carried out in accordance with the Chinese specifications of the Test Code for Hydraulic Asphalt Concrete, DL/T 5362-2018 [21].

In this paper, the preparation of the hydraulic asphalt concrete consisted of coarse and fine limestone aggregate, filler and hydraulic bitumen. The combined aggregate gradation was determined by using Dingburong’s equation, as proposed in Chinese specification [6]:

(3) $$P_{i}=P_{0.0075}+(100-P_{0.075})\frac{{\left(d_{i}\right)}^{r}-0.075^{r}}{D^{r}-0.075^{r}}$$

where:

*P_i_* = Percent passing at the sieve size of *d_i_*;
*D* = The maximum grain size of aggregates in millimeters, which equals 16 mm;
*d_i_* = Sieve size, mm;
*R* = Gradation index;
*P*_0.075_ = Filler content, percent passing at the sieve size of 0.075 mm.

Based on the engineering experience of the asphalt concrete impermeable layer of PSPSs in China, the commonly accepted and used gradation index was around 0.4 together with 9% filler content. A bitumen–aggregate ratio of 7.6% was commonly used, that is, the corresponding bitumen content was 7.06%. In order to simplify the concrete test plan, only one mix proportion of the hydraulic asphalt mixture was considered. This mix design was determined by a combination of *D* = 16 mm, *r* = 0.4, *P*_0.075_ = 9% and a bitumen content 7.06%. The combined aggregate gradation is given in Table 2.

**Figure 1:**
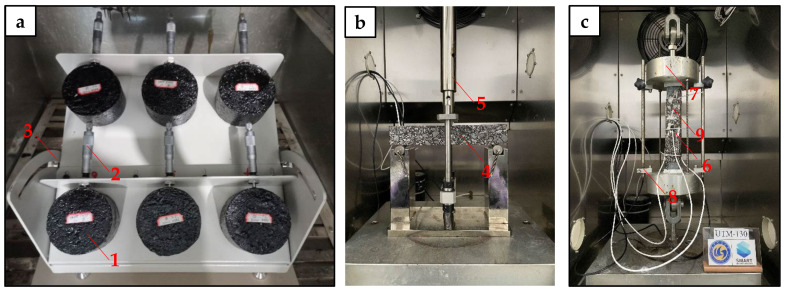
Setups of asphalt concrete slope flow test (**a**), three-point bending (3PB) test (**b**) and thermal stress restrained specimen test (TSRST) (**c**), where: 1—Marshall specimen; 2—Micrometer; 3—Tilt adjustable oblique panel.; 4—3PB specimen; 5—Loading device; 6—Temperature transducer; 7—Cohesive devices; 8—Displacement transducer; 9—TSRST specimen.

The slope flow test setup of the hydraulic asphalt concrete is shown in Figure 1a. Each group consisted of six Marshall specimens and all of them were fixed on the test platform with a slope of 1:1.7. The whole test setup was kept in a chamber at a temperature of 70 °C for 48 h. After that, the slope flow value was measured by a spiral micrometer at the location specified as 50 mm height from the bottom of each specimen.

The three-point bending (3PB) test was performed by a UTM-130 test machine to evaluate the flexibility of the hydraulic asphalt concrete at low temperatures and the used setup is shown in Figure 1b. The size of the test specimen’s beam was 250 mm × 40 mm × 35 mm. Before testing, the specimens were kept in a chamber of 2 °C for at least 4 h. The 3PB test was done at a loading rate of 0.5 mm/min.

The thermal cracking resistance of the hydraulic asphalt concrete at extremely low temperature was assessed by means of the thermal stress restrained specimen test (TSRST) method [27]. The TSRST test was carried out by a UTM-130 test machine and the used setup is shown in Figure 1c. The size of the test specimen’s beam was 200 mm × 40 mm × 40 mm. During testing, the two ends of test specimen were fixed to obtain a constant length, while the temperature was controlled to decrease at a rate of 30 °C/h, with an initial temperature of 10 °C. As the temperature decreases, the specimen will shrink and result in internal tensile stress. The specimen will fracture at a critical temperature when the subjected tensile stress exceeds the tensile strength. The fracture temperature of hydraulic asphalt concrete is defined at the temperature when the test specimen breaks. Similarly, the fracture strength is indicated by the maximum stress at failure. It should be noted that lower fracture temperature together with lower fracture strength is desired for hydraulic asphalt concrete with better thermal cracking resistance.

## 3. Results and Discussion

### 3.1. DSR Test Results

#### 3.1.1. Master Curves and Black Diagrams of Rheological Properties

The complex modulus (G*) and phase angle (δ) were obtained from the DSR frequency sweep tests at various temperatures. A master curve was constructed for each type of hydraulic bitumen based on the time–temperature superposition. In this study, the reference temperature was selected as 20 °C. Frequency sweep test data at other temperatures were shifted horizontally to obtain a smooth master curve of the complex modulus as well as the phase angle [28]. The shift factors at various temperatures were usually described by using the Arrhenius equation [29].

(4) $$log\left(\alpha_{T}\right)=\frac{\Delta E_{a}}{2.303R}\left(\frac{1}{T}-\frac{1}{T_{0}}\right)$$

where:

*α_T_* = Temperature shift factor;
*T* = Test temperature, K;
Δ*Ea* = Apparent activation energy, kJ/mol;
*R* = Universal gas constant, *R* = 8.314 J/(K·mol).

The master curve of the complex modulus and the phase angle constructed at a reference temperature is usually a function of the reduced frequency. In general, the master curve can be mathematically modelled by a sigmoidal-shape function, described as follows [30,31]:

(5) $$G^{*}=G_{e}^{*}+\frac{G_{g}^{*}-G_{e}^{*}}{{\left[1+{\left(f_{c}/f^{\prime }\right)}^{k}\right]}^{m_{e}/k}}$$

where:

$G_{e}^{*}$ = $G^{*}\left(f\to 0\right)$, equilibrium complex modulus, $G_{e}^{*}=0$;
$G_{g}^{*}$ = $G^{*}\left(f\to \infty \right)$, glass complex modulus, Pa;
f_c_ = Location parameter with dimensions of frequency, Hz;
$f^{\prime }$ = Reduced frequency, function of temperature, Hz;
k, m_e_ = Shape parameters, dimensionless.

The reduced frequency is determined by multiplying the test frequency by the shift factor, α_T_:

(6) $$f^{\prime }=f\times \alpha_{T}$$

The equation for the master curve of the phase angle is as follows:

(7) $$\delta =90I-\left(90I-\delta_{m}\right){\left\{1+{\left[\frac{\log\left(f_{d}/f^{\prime }\right)}{R_{d}}\right]}^{2}\right\}}^{-m_{d}/2}$$

where:

$\delta_{m}$ = Phase angle constant at f_d_, the maximum value for asphalt mixtures and the value at the inflexion for asphalt binders;
$f^{\prime }$ = Reduced frequency;
f_d_ = Location parameters with dimensions of frequency, at which $\delta_{m}$ occurs, Hz;
$R_{d}$, $m_{d}$ = Shape parameters; $I=\left\{\begin{array}{c} 0\;\mathrm{for}\;\mathrm{mixtures}\\ \left\{\begin{array}{c} 0\;\mathrm{if}\;f>f_{d}\\ 1\;\mathrm{if}\;f\leq f_{d} \end{array}\right. \end{array}\right.\mathrm{for}\;\mathrm{binders}$.

The experimental data obtained from the DSR testing were then fitted to the sigmoidal-shape functions given by Equations (5) and (7), respectively. All of the model parameters or constants can be automatically obtained by minimizing the mean relative error using the Solver function in the Excel spreadsheet. The determination of the model parameters for the master curves are listed in Table 3. As indicated by the value of R^2^, good fit was obtained for the prediction of the master curves as well as the shift factor. However, it should be noted that the Arrhenius equation has its limit to fit at a wide temperature range between −10 °C and 70 °C. In particular, a slight deviation between the model prediction and the actual value was found at −10 °C [29].

As can be seen in Table 3, the Jurong SG90 bitumen had the maximum value of the glass complex modulus, followed by Karamay SG70, Jingbo SG70 and Liaohe SG90 bitumen; Jingbo PMB showed the minimum value. This indicated that the SBS polymer modification had a positive effect to reduce the bitumen’s brittleness at extreme low temperature.

With respect to the phase angle constant δ_m_, it reflected the ratio between the viscosity and elasticity of bitumen at low reduced frequency, which corresponded to high temperature. The lower value of δ_m_ indicates a higher fraction of elasticity, which was desired for thermal stability and creep resistance. Among these five hydraulic bitumen binders, Jingbo PMB showed the minimum value, following by Liaohe SG90, Jurong SG90 and Jingbo SG70; Karamay SG70 bitumen had the maximum value of δ_m_. This also indicated that the SBS polymer modification had a positive effect to improve the bitumen’s performance at high temperature. The apparent activation energy Δea usually relates to the temperature sensitivity of bitumen. The data listed in Table 3 showed that the value of Δ*ea* had a narrow range between 173 and 178 kJ/mol, indicating a similar temperature sensitivity for all of the five bitumen binders.

**Figure 2:**
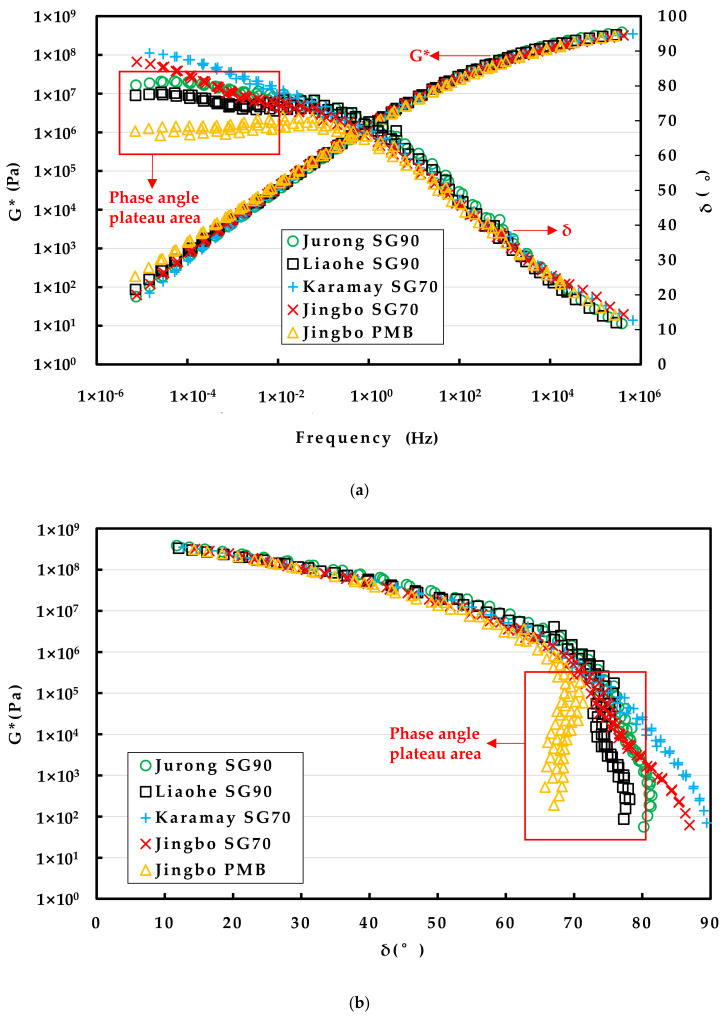
The master curves of complex modulus and phase angle of five hydraulic bitumen binders (**a**) and black diagrams (**b**).

Figure 2a shows the master curve of the complex modulus and the phase angle of the five hydraulic bitumen binders. In this figure, it can be observed that the complex modulus increased as the frequency increased for all of the hydraulic bitumen binders. Because high frequency corresponded to low temperature, it was found that the descending ranking of the complex modulus at low temperature was as follows: Jurong SG90 hydraulic bitumen, Karamay hydraulic bitumen, Liaohe SG90 hydraulic bitumen, Jingbo SG70 hydraulic bitumen and Jingbo PMB. With respect to high temperature, which corresponded to low frequency, the descending ranking of complex modulus at high temperature was as follows: Jingbo PMB, Karamay SG70 hydraulic bitumen, Liaohe SG90 hydraulic bitumen, Jingbo SG70 hydraulic bitumen and Jurong SG90 hydraulic bitumen.

Similar to the complex modulus, it can be observed that the phase angle decreased as the frequency increased for all of the hydraulic bitumen binders. Among these five bitumen binders, the Jingbo SG70 hydraulic bitumen, Jurong SG90 hydraulic bitumen and Liaohe SG90 hydraulic bitumen exhibited an obvious change when compared with Karamay SG70. A remarkable plateau was seen for Jingbo PMB. The change of the phase angle master curve, especially the plateau, usually indicated the existence of the polymer. Because the degree of change strongly depends on the polymer content, it can be inferred that the Jingbo SG70 hydraulic bitumen, Jurong SG90 hydraulic bitumen and Liaohe SG90 hydraulic bitumen were slightly modified by a relatively small amount of polymer when compared with Jingbo PMB, which usually has a polymer content of around 4%.

Figure 2b shows the black diagrams based on the complex modulus and the phase angle. The curves exhibited the relationship between the measured complex modulus and the phase angle on a semi-logarithmic scale. It can be observed that the complex modulus decreased as the phase angle increased for all of the hydraulic bitumen binders. Karamay SG70 bitumen exhibited a smooth decline tendency over a wide range of phase angles. However, the Jingbo SG70 hydraulic bitumen, Jurong SG90 hydraulic bitumen and Liaohe SG90 hydraulic bitumen exhibited a slight deviation from the smooth trend. A distinct deviation can be found for Jingbo PMB. The complex modulus dropped dramatically as the phase angle remained in a plateau between 60° and 70°. It should be noted that the existence of the phase angle plateau is believed to be attributed to the addition of polymer into bitumen. Again, the degree of the plateau demonstrated that the Jingbo SG70 hydraulic bitumen, Jurong SG90 hydraulic bitumen and Liaohe SG90 hydraulic bitumen were slightly modified by a smaller amount of polymer when compared with real Jingbo PMB.

The above analysis demonstrated that the DSR phase angle master curves and black diagrams gave more insights than the results obtained from the traditional penetration, softening point and ductility. The DSR test can well distinguish the existence of the SBS polymer in the Jurong SG90 hydraulic bitumen, Liaohe SG90 hydraulic bitumen and Jingbo SG70 hydraulic bitumen. This indicated that the addition of the SBS polymer and the change of bitumen can be detected, and thus make the DSR test a powerful method for the purpose of the quality control of bitumen.

#### 3.1.2. Complex Modulus (G*) and Phase Angle (δ) at 70 °C

**Figure 3:**
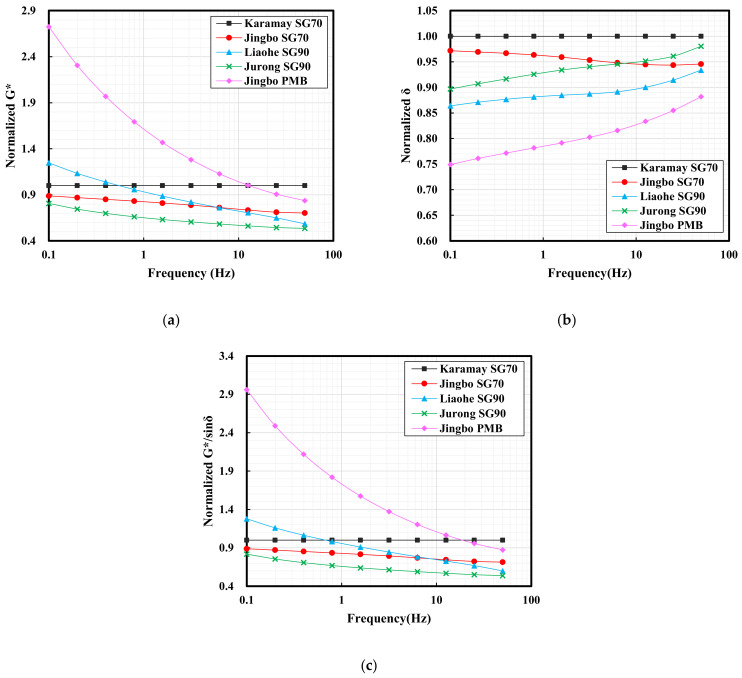
The normalized complex modulus (**a**), phase angle (**b**) and G*/sinδ (**c**) of five hydraulic bitumen binders at 70 °C.

Table 4 and Figure 3 show the complex modulus and phase angles of five hydraulic bitumen binders at 70 °C. For an ideal bitumen binder which has good high-temperature performance, a higher complex modulus and a lower phase angle at high temperatures are needed. In the field of road paving bitumen, a rutting factor, which is defined as G*/sinδ, is widely used to evaluate bitumen’s high-temperature performance. As can be seen, G*/sinδ makes good use of a combination of G* and sinδ. Higher G* together with lower δ will result in higher G*/sinδ, and thus an improved high-temperature performance. For this reason, the data obtained from G*/sinδ were also evaluated in Figure 3.

As can be seen in Table 4, all the bitumen binders exhibited an increase on G* with a reduction on δ as the test frequency increased. These changes seemed to be dependent on the type of bitumen. The Karamay SG70 bitumen had a phase angle of 89.4, indicating that it is close to Newtonian fluid at 70 °C. For the ease of comparison, the Karamay SG70 bitumen was selected as the reference bitumen, since it is commonly accepted and used as a hydraulic bitumen in China. The G* and δ obtained from other bitumen binders were normalized using the Karamay SG70 bitumen as the reference value of 1 at various frequencies. To do this, the G* and δ of other bitumen binders were divided by the corresponding value of the Karamay SG70 bitumen and the obtained ratios were illustrated in Figure 3. Similarly, the values of G*/sinδ were also normalized.

At high temperature, the slope flow is the main risk for the hydraulic asphalt concrete impermeable layer. The slope flow is a long-term behavior, and thus the rheological properties at low frequency are of interest. In this case, Jingbo PMB showed an overall higher G*, followed by Liaohe SG90 bitumen with a slight improvement on G* when the frequency was lower than 0.5 Hz. The rest of the bitumen binders exhibited lower G*. With respect to the normalized δ, all the bitumen binders showed lower values compared to the Karamay SG70 bitumen. Significant reduction can be seen on Jingbo PMB as well as the Liaohe SG90 bitumen. The normalized values of G*/sinδ were similar to those of the normalized G* for all of the bitumen binders. The analysis above indicated that Jingbo PMB was superior to the other bitumen binders with respect to high-temperature properties.

When using the complex modulus, the phase angle and G*/sinδ as the indicators for high-temperature performance, Jingbo PMB was superior to other bitumen binders, indicated by the higher complex modulus, the lower phase angle as well as the higher G*/sinδ. Some hydraulic bitumen binders that were slightly modified by the SBS polymer did not always show consistent results when the traditional Karamay hydraulic bitumen was used as a reference.

#### 3.1.3. Complex Modulus (G*) and Phase Angle (δ) at −10 °C

**Figure 4:**
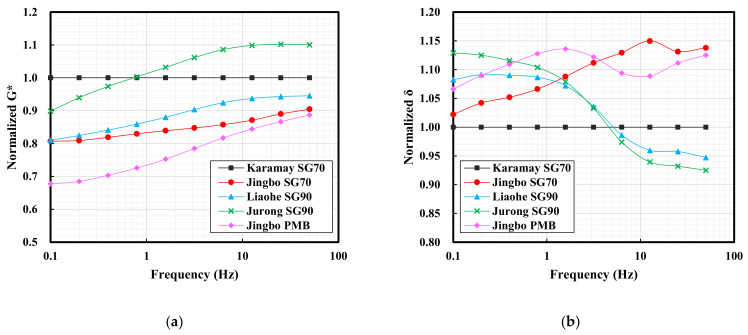
The complex modulus (**a**) and phase angle (**b**) of five hydraulic bitumen binders at −10 °C.

Table 5 and Figure 4 show the complex modulus and the phase angle of the five hydraulic bitumen binders at −10 °C. The large flexibility at low temperatures is related to the lower complex modulus and the higher phase angle. The rheological characteristics of the bitumen (the complex modulus and phase angle) showed a strong relationship to the fracture temperature of asphalt mixtures. It was reported that bitumen with a phase angle smaller than 15° and a complex modulus higher than 300 MPa was more likely to fracture at low temperature [32,33].

As can be seen in Table 5, all the bitumen binders exhibited an increase on G* with a reduction on δ as the test frequency increased. With a frequency higher than 25 Hz, some bitumen binders tended to have a complex modulus higher than 300 MPa together with a phase angle lower than 15°. This indicates that the rapid temperature drop is decisive for thermal cracking at low temperature. Similarly, the G* and δ were normalized using Karamay SG70 bitumen as the reference, as mentioned before, and the obtained results were presented in Figure 4.

At extreme low temperature, the thermal shrinkage cracking is the main risk for the hydraulic asphalt concrete impermeable layer. The cracking resistance strongly dependent on the degree of temperature, the drop rate as well as the bitumen flexibility. After normalization, it can be clearly seen that the other bitumen binders tended to have low G* compared to the Karamay SG70 bitumen. An exception was found on the Jurong SG90 bitumen at frequencies higher than 1 Hz. With respect to the normalized δ, all the bitumen binders tended to have higher values compared to the Karamay SG70 bitumen. An exception was found on the Jurong SG90 bitumen and the Liaohe SG90 bitumen at frequencies higher than 8 Hz. In general, Jingbo PMB showed the lowest G* combined with a relatively high δ among the five bitumen binders. In general, when using the complex modulus and the phase angle as the indicators for low temperature performance, Jingbo PMB was superior to the other bitumen binders with respect to the flexibility at low temperature. Similar to the high temperature performance, as discussed before, some hydraulic bitumen binders that were slightly modified by the SBS polymer did not always show consistent results when the traditional Karamay hydraulic bitumen was used as a reference. This indicates the complexity of the system of slightly modified bitumen, and thus the stability may become a big concern.

### 3.2. FTIR Results

As indicated by the master curve of the phase angle and the black diagram, it was deduced that there may be the existence of polymer in the Jingbo SG70 bitumen, Jurong SG90 bitumen and Liaohe SG90 bitumen. To further confirm this point, FTIR spectrum analysis was carried out on all of the five bitumen binders. For the purpose of comparison, the SBS polymer was also involved to well reflect the characteristic absorption peaks at 966 cm^−1^ and 699 cm^−1^, which corresponded to the functional groups of the polybutadiene (PB) and polystyrene (PS).

**Figure 5:**
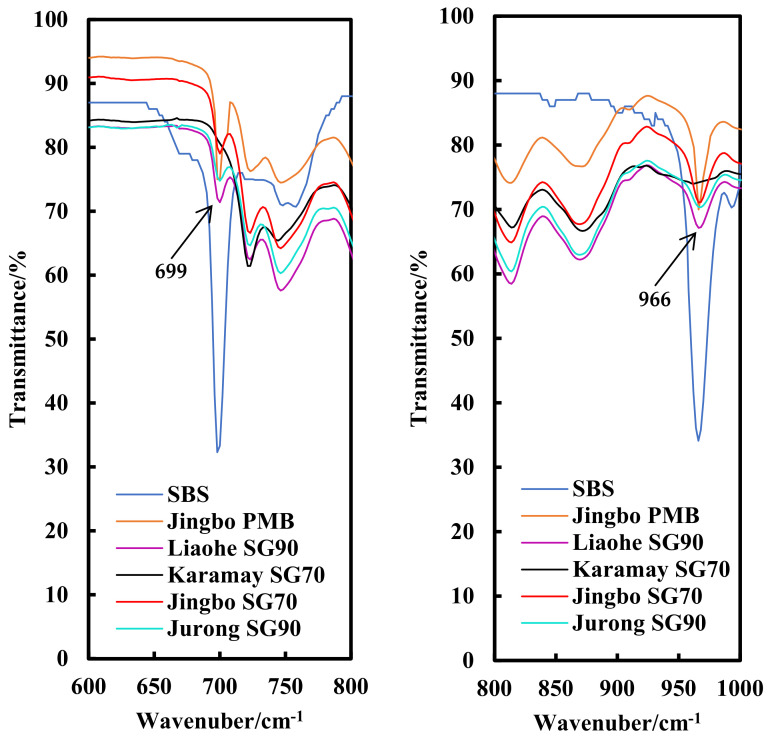
Infrared spectra of SBS and hydraulic bitumen binders.

As can be seen from Figure 5, the SBS polymer showed strong peaks at 966 cm^−1^ and 699 cm^−1^, while there were no peaks for the Karamay SG70 bitumen, indicating that no SBS polymer was added. However, the Jingbo SG70 bitumen, Jurong SG90 bitumen and Liaohe SG90 bitumen also exhibited small peaks at 966 cm^−1^ and 699 cm^−1^. The peaks of these three bitumen binders were relatively weaker than those of Jingbo PMB. Therefore, it can be concluded that the Jingbo SG70 bitumen, Jurong SG90 bitumen and Liaohe SG90 bitumen were slightly modified by small amount of SBS polymer. The results obtained from the FTIR spectra were consistent with the DSR rheological data, as discussed earlier.

### 3.3. Multiple Stress Creep Recovery (MSCR) Test Results

**Figure 6:**
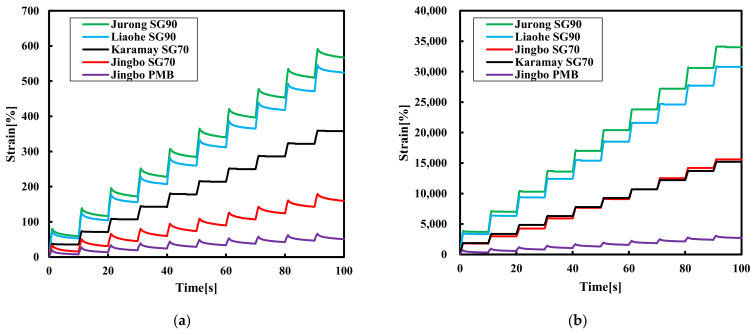
The MSCR results of the five bitumen binders at (**a**) 0.1 kPa and (**b**) 3.2 kPa.

Figure 6 displays the MSCR test results of the five bitumen binders at two shear stress levels of 0.1 and 3.2 kPa. When the Karamay SG70 bitumen was selected as the reference bitumen, it can be seen that the recovery after the unloading was negligible at 0.1 kPa. Very limited recovery was also observed for the Jurong SG90 bitumen and Liaohe SG90 bitumen. However, the Jingbo SG70 bitumen had a slight creep recovery, while Jingbo PMB had a significant recovery. As the stress level increased to 3.2 kPa, the creep recovery reduced for all the bitumen binders. This strongly indicated the stress dependency of bitumen.

Table 6 presents the results of the non-recoverable compliance and recovery percentage of five bitumen binders at 70 °C. It should be noted that a lower non-recoverable creep compliance together with a high recovery percentage was desired for bitumen with better creep resistance at high temperature. Similarly, the Karamay SG70 bitumen was selected as the reference bitumen. In this case, it can be seen that the Jurong SG90 bitumen had the highest value of non-recoverable compliance at 0.1 kPa, followed by the Liaohe SG90 bitumen and the Karamay SG70 bitumen. Jingbo PMB had the lowest value among the five bitumen binders. As the stress level increased to 3.2 kPa, the non-recoverable creep compliance increased slightly for the five hydraulic bitumen, and the values of their results were similar to 0.1 kPa.

With respect to the recovery percentage, the Karamay SG70 bitumen showed a limited recovery of 3.7%. An obvious improvement of 27.6%, 27.7% and 54.8% was found for the Liaohe SG90 bitumen, Jurong SG90 bitumen and Jingbo SG70 bitumen, respectively. Obviously, Jingbo PMB had the highest recovery of 74.6% among the five bitumen binders. As the stress level increased to 3.2 kPa, the recovery capacity of the Jingbo SG70 bitumen, Jurong SG90 bitumen and Liaohe SG90 bitumen disappeared and the recovery of Jingbo PMB was reduced to 50.2%.

The above analysis indicated that the recovery percentage was strongly dependent on the effect of the polymer modification. The Karamay SG70 bitumen was a virgin bitumen without polymer modification, and thus there was a negligible recovery. The Jingbo SG70 bitumen, Jurong SG90 bitumen and Liaohe SG90 bitumen were found to be slightly modified by the SBS polymer, and thus the recovery can be expected to be smaller than that of Jingbo PMB. It can be concluded that the hydraulic bitumen binders prepared using traditional road paving bitumen modified with a small amount of the SBS polymer can have some improved properties comparable to the Karamay SG70 bitumen. However, the storage stability, aging resistance and durability of these bitumen binders was questionable. Currently, the application of SBS polymer-modified bitumen is accepted to deal with extreme temperature conditions at which the virgin bitumen usually has its limits.

Compared with the results obtained from the traditional softening point test, it can be concluded that the non-recoverable compliance and recovery percentage obtained from the DSR creep test gave more insights into the high-temperature performance of the different hydraulic bitumen binders. In general, the proper ranking of the various bitumen binders can be obtained, and thus these two parameters can serve as a performance index for the control of the slope flow resistance.

### 3.4. Stress Relaxation Test Results

**Figure 7:**
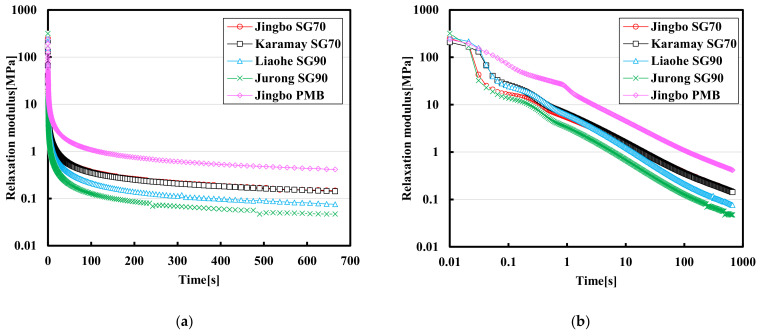
Modulus relaxation curves of semi-log scale (**a**) and log-log scale (**b**) of five hydraulic bitumen binders at −10 °C.

**Figure 8:**
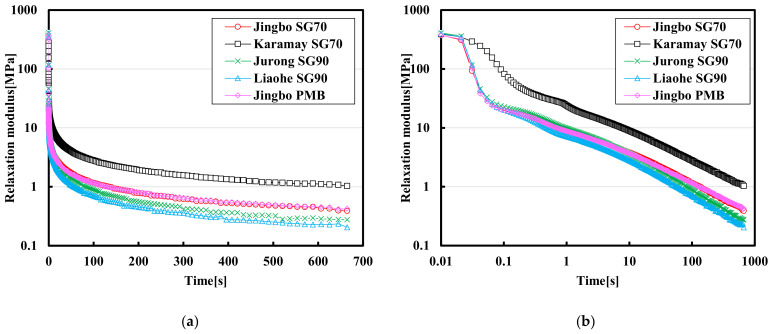
Modulus relaxation curves of semi-log scale (**a**) and log-log scale (**b**) of five hydraulic bitumen binders at −25 °C.

Figure 7 and Figure 8 show the relaxation modulus curves over time at a linear scale, as well as at the log–log scale at −10 °C and −25 °C. The relaxation modulus was calculated using the subjected stress over time divided by the applied constant instantaneous strain. As can be seen, the modulus relaxation was very rapid at the initial state, and a decline of several orders of magnitude was observed. When the relaxation modulus was plotted against time at the log–log scale, a linear relationship can be obtained. During the stress relaxation test, the relation for the time-dependent modulus can usually be explained by using the following formula:

(8) $$E\left(t\right)=\frac{\sigma \left(t\right)}{\varepsilon }=E_{0}\cdot exp\left(-t/\lambda \right)$$

where:

*E*(*t*) = Relaxation modulus, MPa;
σ(*t*) = Relaxation stress, MPa;
*E*_0_ = Instantaneous modulus, MPa;
ε = Instantaneous strain;
*t* = Relaxation time, s;
λ = Model constant, s. λ represents the time in which the modulus relaxes to 1/e or 37% of its initial modulus value *E*_0_.

Equation (8) can be further translated into Equation (9) by using the log–log scale:

(9) $$LogE\left(t\right)=\mathrm{Log}E_{0}+0.4343\times \left(-t/\lambda \right)$$

As indicated in Figure 7a, the residual relaxation modulus of Jingbo PMB is the largest at −10 °C, and that of the Jurong SG90 bitumen is the smallest among these five bitumen binders. The Karamay SG70 bitumen and the Jingbo SG70 bitumen tended to have a very similar behavior in terms of the stiffness reduction. As indicated in Figure 7b, it can be seen that all of these five bitumen binders followed a similar slope in the log–log scale, indicating their rate of modulus relaxation was close to each other.

As the temperature was reduced to −25 °C, the Karamay SG70 bitumen had the largest residual relaxation modulus, and the Liaohe SG90 bitumen had the smallest residual stiffness modulus. The difference between the Jurong SG90 bitumen and the Liaohe SG90 bitumen was small. The same phenomenon was also found between the Jingbo SG70 bitumen and Jingbo PMB. It seemed that the bitumen binders obtained from the same producer tended to have similar relaxation behaviors at extreme low temperature.

Table 7 and Table 8 present the relaxation modulus at some specific times (including 0.1 s, 10 s and 600 s) as well as the fitting results of Equation (8) on various bitumen binders. At −10 °C, the value of E_0_ ranged from 205.72 MPa to 303.79 MPa after 0.1 s, while the modulus reduced to a range between 14.13 MPa and 75.82 Mpa. As the relaxation time was further increased to 10 s, the modulus reduced to a range between 0.68 MPa and 4.63 MPa. Finally, the relaxation modulus ranged between 0.05 MPa and 0.44 MPa with a relaxation time of 600 s.

As the temperature was reduced from −10 °C to −25 °C, the relaxation modulus was increased, indicating the residual stress was also increased. However, the rate of the modulus relaxation at −25 °C was also very rapid. For example, the value of E_0_ ranged from 372.29 Mpa to 410.49 Mpa. After 0.1 s, the modulus was reduced to a range between 19.87 Mpa and 82.10 Mpa. As the relaxation time was further increased to 10 s, the modulus was reduced to a range between 2.78 MPa and 8.87 MPa. Finally, the relaxation modulus ranged between 0.23 MPa and 1.10 MPa with a relaxation time of 600 s. The final value of the relaxation modulus at the end usually related to the risk of the residual stress that may result in thermal shrinkage cracking. As indicated, a drop of temperature from −10 °C to −25 °C significantly increased the final relaxation modulus and thus the risk of thermal cracking. In this case, the Karamay SG70 bitumen seemed to have a higher risk for thermal cracking at extremely low temperature when compared with the other bitumen binders. The benefit of Jingbo PMB was not so obvious when compared with the rest of the bitumen binders at −25 °C. In general, the modulus relaxation behavior can be well explained by Equation (8). The relaxation rate and the final residual stress would be used as the key indices to evaluate the performance at low temperatures.

### 3.5. Derect Tensile (DT) Test Results

**Figure 9:**
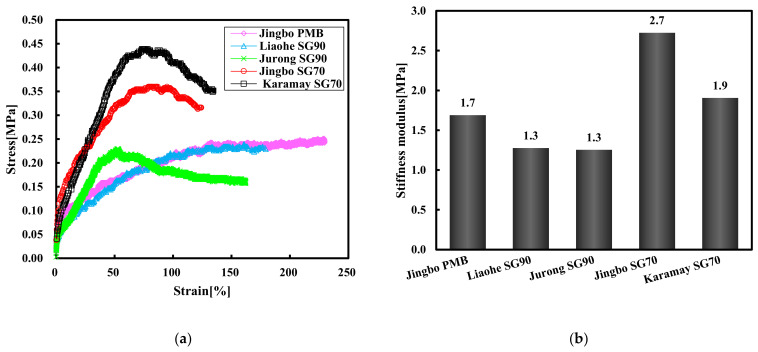
Tensile stress–strain curve (**a**) and stiffness modulus (**b**) of five bitumen binders at 0 °C.

Figure 9 shows the tensile stress–strain curves and the stiffness modulus of the five hydraulic bitumen binders at 0 °C. As indicated by the stress–strain curves, three types of bitumen showed peak values, while the other two types of bitumen binders exhibited ductile failure without peak value. From the point of view of the tensile strain corresponding to the maximum stress, the Jurong SG90 bitumen exhibited the smallest tensile strain, followed by the Karamay and Jingbo SG70 bitumen, and the Liaohe SG90 bitumen and Jingbo PMB showed the greatest failure strain. With respect to the stiffness modulus, the Jingbo SG70 bitumen had the largest modulus, followed by the Karamay SG70 and Jingbo PMB. The Jurong and Liaohe SG90 bitumen had the smallest stiffness modulus. In general, Jingbo PMB showed an excellent ductility and the other slightly modified bitumen also had improved ductile properties when compared to Karamay SG70.

**Figure 10:**
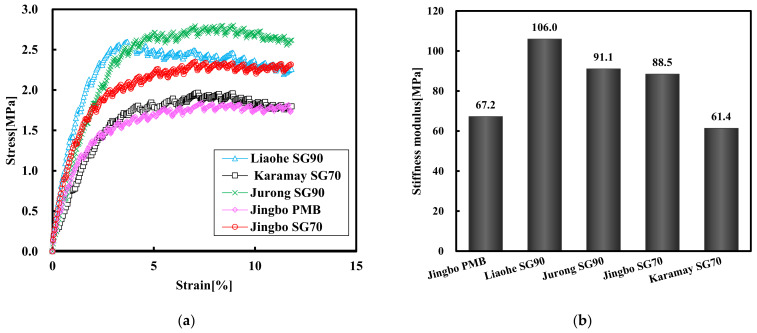
Tensile stress–train curve (**a**) and stiffness modulus (**b**) of five bitumen binders at −15 °C.

Figure 10 shows the tensile stress–strain curves and the stiffness modulus of the five hydraulic bitumen binders at −15 °C. At −15 °C, all of the five hydraulic bitumen binders exhibited ductile failure, and thus had the maximum stress strains larger than 10%. With respect to the stiffness modulus, the Liaohe SG90 bitumen had the largest stiffness, followed by the Jurong SG90 and Jingbo SG70 bitumen. The Jingbo PMB and Karamay SG70 bitumen had the smallest stiffness modulus. In general, the Karamay SG70 bitumen showed very similar properties with Jingbo PMB. This indicated the former had a superior property compared to the rest of the bitumen binders.

**Figure 11:**
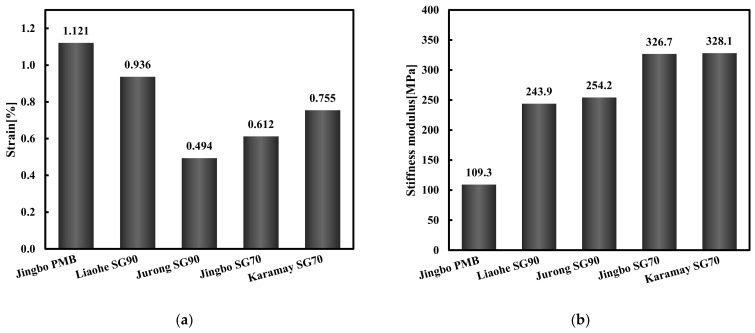
Tensile fracture strain (**a**) and stiffness modulus (**b**) of five hydraulic bitumen binders at −25 °C.

Figure 11 shows the tensile strain at failure and the stiffness modulus of the five hydraulic bitumen binders at −25 °C. Under the test condition of −25 °C, all five hydraulic bitumen binders showed brittle failure. The failure strain of Jingbo PMB was the largest, followed by the Liaohe SG90 bitumen, the Karamay bitumen and the Jingbo SG70 bitumen; the Jurong SG90 bitumen had the smallest failure strain. From the analysis of the stiffness modulus, it can be seen that Jingbo PMB was the smallest, followed by the Liaohe SG90 bitumen and the Jurong SG90 bitumen. Both the Jingbo SG70 bitumen and the Karamay bitumen had a relatively high stiffness modulus.

Compared with the Karamay SG70 bitumen, only Jingbo PMB showed a good crack resistance at low temperatures. However, the slightly modified hydraulic bitumen did not show a consistent performance at different low temperatures. In particular, at −25 °C the benefit of a slight modification was relatively limited. Based on the knowledge of asphalt pavement, the bitumen with a tensile strain larger than 1% together with a stiffness lower than 300 MPa had a superior performance to resist thermal cracking at extremely low temperature. In this case, only Jingbo PMB can fulfill these two requirements. Both the Karamay SG70 bitumen and the Jingbo SG70 bitumen had lower tensile failure strain together with higher stiffness, indicating that both of the bitumen binders could be susceptible to thermal cracking. In general, the tensile fracture strain and the stiffness modulus are good indicators for the brittleness of bitumen at extremely low temperatures. Therefore, it was proposed to establish to the critical values for the tensile fracture strain and the stiffness modulus to reduce the risk of brittle crack.

### 3.6. Test Results of Various Asphalt Mixtures

Table 9 shows the summary results obtained from the slope flow test, the 3PB test and the TSRST for various hydraulic asphalt mixtures. Three typical hydraulic bitumen binders were considered, including the Karamay SG70 bitumen, the Jingbo SG70 bitumen and Jingbo PMB. The selected bitumen binders represented virgin bitumen, slightly- and normal-polymer-modified bitumen. It should be noted that all of the asphalt mixtures were prepared with the same mixture proportion but using different bitumen binders.

When the mixture proportion was constant, the high-temperature slope flow of the hydraulic asphalt concrete was mainly related to the high-temperature creep resistance of the bitumen. The creep performance of the bitumen was characterized by Jnr and %recovery. Lower Jnr and higher %recovery indicated better creep resistance, and thus an expected lower slope flow for the hydraulic asphalt concrete. As indicated in Table 9, Jingbo PMB showed the smallest slope flow value, followed by the Jingbo SG70 bitumen, and the Karamay SG70 bitumen had the largest slope flow value. This tendency was in agreement with the results of the bitumen creep test. It indicated that the bitumen creep test can be used for the purpose of the optimization selection of raw materials.

The 3PB test was used to evaluate the flexibility of the hydraulic asphalt concrete under the settlement deformation of the subgrade. As indicated in Table 9, the bending flexibility of the hydraulic asphalt concrete related well with the bitumen tensile failure strain at 0 °C by using DT. The Jingbo PMB mixture showed the highest bending strain at failure, which was consistent with the largest tensile failure strain of Jingbo PMB. Jingbo SG70 bitumen did not show its slight modification effect on the mixture bending strain when compared to the Karamay SG70 bitumen. Because of the good correlation between the 0 °C tensile test of the bitumen and the three-point bending test of the asphalt mixture, the bitumen tensile test can also serve as a tool for bitumen optimization.

The bitumen direct tensile test and the stress relaxation at −25 °C, and the TSRST of the asphalt concrete, were used to investigate the relationship between the bitumen and mixture performance at extremely low temperature. As indicated in Table 9, the failure stress of the TSRST for these three types of asphalt concrete was well related to the final relaxation modulus of the used bitumen. However, the failure temperature of the TSRST seemed to be dependent on the bitumen direct tensile strain at −25 °C. In general, the failure temperature of the Jingbo SBS asphalt mixture was lowest, followed by Karamay SG70 and Jingbo SG70. It can be seen that the crack resistance of the asphalt concrete was well related to the bitumen stiffness relaxation rate, as well as the direct tensile strain at failure.

## 4. Conclusions

In this study, a rheological evaluation by means of the DSR and DT tests was performed on five different types of hydraulic bitumen binders obtained from various PSPS projects. The DSR rheological test, creep test, stress relaxation test and DT test were carried out on various bitumen binders. Furthermore, the slope flow test, three-point bending test and thermal stress restrained specimen test were also carried out on hydraulic asphalt mixtures. Based on the experimental results and the analysis done, the following conclusions were drawn:

(1) The remarkable plateau of the phase angle master curves and the black diagrams indicated the existence of the SBS polymer for several penetration-grade hydraulic bitumen binders. Infrared spectra analysis further confirmed these bitumen binders contained a relatively small content of the SBS polymer compared with the traditional SBS-modified bitumen. It was concluded that a slightly modified polymer bitumen had been applied at least for several pumped storage reservoirs, since this type of bitumen is not formally recognized in China.
(2) The DSR rheological master curves of the various hydraulic bitumen binders can be well fitted by using the presented sigmoidal-shape functions. This allowed the establishment of a benchmark for the selection and development of better hydraulic bitumen based on the rheological performance. The DSR master curves test, creep test, stress relaxation test and direct tensile test can be used to well evaluate the performance of hydraulic bitumen.
(3) Hydraulic bitumen binders produced by slightly modified SBS polymer may provide nice data in terms of traditional data on penetration, the softening point and the ductility, but may not really improve the performance. Since the SBS-modified bitumen has both a good low- and high-temperature performance, one can foresee that its application is more promising in the future, especially when dealing with extreme service temperatures.
(4) The attempt to establish a relation between the bitumen and mixture performance by using the bitumen creep, relaxation, and tensile failure strain corresponding to the asphalt concrete slope flow, maximum bending strain and failure temperature, respectively, was made. It was found that the bitumen performance can well explain the performance of the prepared hydraulic asphalt concrete. It indicated that the bitumen creep test, direct tensile test and stress relaxation test can be used for the optimization of bitumen.

The purpose of this study was to propose an evaluation method for hydraulic bitumen based on rheological properties. The evaluation methods and control indices of the high-temperature creep properties and the low-temperature limit strains of the hydraulic bitumen were put forward. It was aimed to guide the mixture design for an impermeable layer with a better high-temperature slope flow and low-temperature cracking resistance. The relation between the bitumen and concrete performance was not yet established to realize the optimal design and performance control of the asphalt mixture based on the bitumen optimization. In the future, more studies will be needed to reduce the gap between the bitumen properties and the real mixture performance. The limit of non-recoverable compliance at 70℃ and the tensile strain at extremely low temperature should be established as the performance-based control indices for the purpose of bitumen optimization. Furthermore, the effects of short-term and long-term aging should also be investigated.

## Figures and Tables

**Table 1:** Basic properties of five hydraulic bitumen binders.

Major Indices		Technical Requirement			Type of Bitumen				
		SG70	SG90	SBS (I-C)	Karamay SG70	Jingbo SG70	Jurong SG90	Liaohe SG90	Jingbo PMB
25 °C Penetration/0.1 mm		61~80	81~100	60~80	69	65	90	88	66
Softening point/°C		47~55	45~52	≥55	49.0	52.0	48.6	51.9	59.5
4 °C ductility/cm		≥10	≥30	≥30 *	23.0	82.0	80.0	78.4	36.0 *
After TFOT(163 °C, 5 h)	Mass loss/%	≤0.4	≤0.6	≤1.0	−0.100	−0.108	−0.132	−0.134	−0.151
	Penetration ratio/%	≥68	≥70	≥60	81	71	73	76	72
	4 °C ductility/cm	≥4	≥8	≥20 *	11.0	17.0	38.5	50.5	22.0 *
	Softening point increment/°C	≤5	≤5	≤5	3.5	3.5	2.0	−2.0	−3.5

Note: * indicates the ductility test of the polymer-modified bitumen was done at loading rate of 5 cm/min and temperature of 5 °C.

**Table 2:** Combined aggregate gradation of hydraulic asphalt concrete.

Sieve Size (mm)	16	13.2	9.5	4.75	2.36	1.18	0.6	0.3	0.15	0.075	Bitumen Content/%
Passing percentage (%)	100	92.4	80.6	60.3	44.9	33.3	24.7	17.9	12.9	9.0	7.06

**Table 3:** Determination of model parameters for master curves.

Model Parameters		Karamay SG70	Jingbo SG70	Jurong SG90	Liaohe SG90	Jingbo PMB
Complex modulus	$G_{e}^{*}$	0				
	$G_{g}^{*}$	6.80 × 10^8^	6.11 × 10^8^	6.93 × 10^8^	5.46 × 10^8^	5.60 × 10^8^
	*f_c_*	130.914	209.347	472.563	578.571	1428.173
	*m_e_*	0.991	0.928	0.906	0.858	0.780
	*k*	0.219	0.228	0.258	0.279	0.276
	R^2^	1.000	1.000	1.000	0.999	0.999
Phase angle	$\delta_{m}$	81.987	77.352	75.869	73.352	68.545
	*f_d_*	0.001	0.003	0.053	0.047	0.031
	*R_d_*	99.581	98.115	96.614	99.356	320.772
	*m_d_*	479.553	484.488	736.000	775.344	6724.741
	R^2^	0.988	0.987	0.995	0.997	0.979
Activation energy	Δ*E_a_*	173.054	177.669	178.257	176.504	175.334
	R^2^	0.991	0.995	0.995	0.996	0.996

**Table 4:** The complex modulus (G*) and phase angle (δ) of five hydraulic bitumen binders at 70 °C.

Frequency/Hz	Karamay SG70		Jingbo SG70		Liaohe SG90		Jurong SG90		Jingbo PMB	
	G*/kPa	δ/°	G*/kPa	δ/°	G*/kPa	δ/°	G*/kPa	δ/°	G*/kPa	δ/°
0.1	0.070	89.4	0.062	86.9	0.087	77.3	0.056	80.2	0.190	67.0
0.2	0.139	89.0	0.120	86.3	0.157	77.6	0.103	80.8	0.319	67.8
0.4	0.274	88.4	0.233	85.5	0.285	77.5	0.192	81.0	0.540	68.2
0.8	0.539	87.5	0.448	84.3	0.517	77.1	0.357	81.0	0.914	68.4
1.6	1.053	86.5	0.854	82.9	0.934	76.5	0.665	80.8	1.546	68.4
3.2	2.037	85.3	1.604	81.3	1.672	75.7	1.234	80.2	2.610	68.5
6.4	3.902	84.1	2.972	79.7	2.968	74.9	2.275	79.5	4.401	68.6
12.8	7.406	82.7	5.436	78.1	5.228	74.4	4.164	78.7	7.433	68.9
25.0	13.937	81.4	9.909	76.8	9.068	74.4	7.592	78.2	12.643	69.6
50.0	26.210	80.0	18.401	75.7	15.366	74.7	14.031	78.5	21.931	70.6

**Table 5:** The complex modulus (G*) and phase angle (δ) of five hydraulic bitumen binders at −10 °C.

Frequency/Hz	Karamay SG70		Jingbo SG70		Liaohe SG90		Jurong SG90		Jingbo PMB	
	G*/MPa	δ/°	G*/MPa	δ/°	G*/MPa	δ/°	G*/MPa	δ/°	G*/MPa	δ/°
0.1	71.9	36.8	58.0	37.6	58.2	39.9	64.6	41.6	48.7	39.3
0.2	102.1	32.0	82.6	33.4	84.2	35.0	95.9	36.0	69.9	34.9
0.4	131.4	28.5	107.6	30.0	110.5	31.1	128.0	31.8	92.4	31.6
0.8	161.8	25.4	134.2	27.1	139.0	27.6	162.3	28.0	117.4	28.6
1.6	193.2	22.7	162.1	24.7	170.0	24.4	199.3	24.5	145.4	25.8
3.2	224.3	20.6	190.1	23.0	202.6	21.4	238.1	21.3	176.0	23.2
6.4	255.2	18.9	218.9	21.4	235.9	18.7	277.2	18.4	208.6	20.7
12.8	287.4	16.9	250.4	19.4	269.4	16.2	315.7	15.9	242.6	18.4
25.0	320.0	14.6	284.8	16.5	301.7	14.0	352.6	13.6	277.2	16.2
50.0	351.9	12.7	318.3	14.4	332.6	12.0	387.3	11.7	312.1	14.3

**Table 6:** The non-recoverable compliance (Jnr) and %recovery (R) tested at 70 °C.

Bitumen	Stress	Karamay SG70	Jingbo SG70	Jurong SG90	Liaohe SG90	Jingbo PMB
Jnr/kPa^−1^	0.1 kPa	3.58	1.60	5.67	5.25	0.51
	3.2 kPa	4.75	4.88	10.63	9.63	0.85
R/%	0.1 kPa	3.7	54.8	27.7	27.6	74.6
	3.2 kPa	0	0	0	0	58.6

**Table 7:** Relaxation modulus at some specific times and the fitting results for various bitumen binders at −10 °C.

Bitumen	E_0_ (MPa)	E@_0.1s_ (MPa)	E@_10s_ (MPa)	E@_600s_ (MPa)	λ	E@_1s_ (MPa)
Karamay SG70	205.72	14.13	0.77	0.07	0.688	0.30
Jingbo SG70	234.67	20.49	1.46	0.15	0.758	5.47
Jurong SG90	303.79	16.70	0.68	0.05	0.625	3.37
Liaohe SG90	261.49	32.03	1.21	0.08	0.611	6.24
Jingbo PMB	236.73	75.82	4.63	0.44	0.715	18.75

**Table 8:** Relaxation modulus at some specific times and the fitting results for various bitumen binders at −25 °C.

Bitumen	E_0_ (MPa)	E@_0.1s_ (MPa)	E@_10s_ (MPa)	E@_600s_ (MPa)	λ	E@_1s_ (MPa)
Karamay SG70	390.59	82.10	8.87	1.10	0.886	7.69
Jingbo SG70	377.00	21.57	3.80	0.43	0.916	8.24
Jurong SG90	410.49	22.61	3.61	0.29	0.803	6.35
Liaohe SG90	399.00	20.09	2.78	0.23	0.804	6.37
Jingbo PMB	372.29	19.87	3.65	0.45	0.930	8.51

**Table 9:** Test results of hydraulic asphalt mixture and the relation with bitumen performance.

Test	Indicators	Unit	Karamay SG70	Jingbo SG70	Jingbo PMB
Mixture slope flow test	Slope flow value	mm	1.51	0.79	0.71
Bitumen creep test at 0.1 kPa	Jnr	kPa^−1^	3.58	1.60	0.51
	R	%	3.7	54.8	74.6
Mixture 3PB test	Flexural strength	MPa	3.63	4.77	4.12
	Maximum bending strain	%	2.705	2.717	3.433
Bitumen DT at 0 °C	Tensile strain	%	73.45	78.84	223.60
Mixture TSRST	Failure temperature	°C	−35.9	−36.7	−39.8
	Failure stress	MPa	3.23	3.55	4.59
Bitumen stress relaxation test at −25 °C	Final relaxation modulus	MPa	0.07	0.15	0.44
	Relaxation rate	-	0.490	0.474	0.467
Bitumen DT at −25 °C	Tensile strain	%	0.755	0.612	1.121

## Data Availability

The reported results of supporting data can be found on the Web of Science.
